# The role of angiotensin II in cardiovascular disease-induced cancer growth

**DOI:** 10.1186/s40959-026-00495-x

**Published:** 2026-05-01

**Authors:** Benji van Berlo, Pierangela Esposito, Alessandra Greco, Charles X. L. van Assche, Tine Bruyns, Birgit Van Asbroeck, Celine Civati, Ronny Mohren, Berta Cillero-Pastor, Pieter-Jan Guns, Gilles W. De Keulenaer, Vincent F.M. Segers

**Affiliations:** 1 Laboratory of Physiopharmacology, Faculty of Medicine and Health Sciences, Faculty of Pharmaceutical, Biomedical and Veterinary Sciences, University of Antwerp, Universiteitsplein 1, UA CDE D.T.218, Antwerp, B-2610 Belgium; 2 Division M4I – Imaging Mass Spectrometry (IMS), Faculty of Health, Medicine and Life Sciences, Maastricht MultiModal Molecular Imaging Institute, Maastricht University, Universiteitssingel 50, Maastricht, 6229 ER The Netherlands; 3 Department of Rehabilitation Sciences & Physiotherapy, Research Group MOVANT, University of Antwerp, Universiteitsplein 1, Antwerp, B-2610 Belgium; 4 Department of Cell Biology‑Inspired Tissue Engineering, Institute for Technology Inspired Regenerative Medicine, Universiteitssingel 40, Maastricht, 6229 ER The Netherlands

**Keywords:** Cardiovascular disease, Cancer, Angiotensin II, RAAS, AT1

## Abstract

**Background:**

The higher incidence of cancer in patients with cardiovascular disease (CVD) has historically been explained by shared risk factors. Recent studies suggest, however, a causal relationship. Nevertheless, the mechanisms of CVD-induced cancer are incompletely understood. Here, we hypothesize that angiotensin II (ANGII) links CVD and increased cancer growth.

**Objective:**

We investigated the impact of ANGII-induced CVD on cancer growth in vivo, differentiating between a direct effect of ANGII on tumor cells or indirect effects secondary to CVD.

**Methods:**

The effect of ANGII on cancer growth was studied in C57BL/6J mice with cancer. Cancer was either induced by subcutaneous injection of Lewis lung carcinoma (LLC) cells, MC38 colon cancer cells, or by genetic susceptibility (APC_min_ mice). To differentiate between direct and indirect effects of ANGII on cancer growth three strategies were implemented: (i) Evaluating a protocol with and without overlap between ANGII treatment and the injection of tumor cells, (ii) comparing the effect of a high (2000 ng.kg^− 1^.min^− 1^) and low (400 ng.kg^− 1^.min^− 1^) dose of ANGII on intestinal polyp growth in APC_min_ mice and (iii) comparing the impact of ANGII on tumor growth in high (LLC) and low (MC38) angiotensin II receptor type I (AT1) expressing tumor cells.

**Results:**

High dose ANGII-treatment induced left ventricle (LV) hypertrophy and cardiac fibrosis, and enhanced growth of injected tumor cells, but only when LCC tumor cells with high expression of AT1 were used, and when these cells were injected during ANGII treatment. ANGII did not increase cancer growth when LCC cells were injected after halting ANGII treatment, or when MC38 tumor cells with low AT1 levels were used. ANGII also increased the number of intestinal polyps in APC_min_ mice, even at a low dose that did not induce LV hypertrophy or cardiac fibrosis. Lastly, an analysis of publicly available cancer databases showed that AT1 gene copy number variation is increased in most human cancer lines and tumors.

**Conclusion:**

This study indicates that ANGII has direct effects on cancer growth, warranting further research into the role of an activated renin-angiotensin-aldosterone-system (RAAS) as a mechanistic link between CVD and cancer growth in AT1-positive tumors.

**Supplementary Information:**

The online version contains supplementary material available at 10.1186/s40959-026-00495-x.

## Introduction

Cardiovascular diseases (CVD) and cancer are the two main causes of death worldwide [1]. While CVD and cancer are separate diseases, epidemiological studies show a higher incidence of cancer in patients with CVD compared to patients without CVD [2, 3]. This higher incidence has historically been attributed to shared risks factors including genetic predisposition, smoking, aging, diabetes, and obesity. In addition, CVD and cancer share overlapping pathophysiological mechanisms including inflammation, oxidative stress, neurohormonal activation, and immune system dysfunction that could further explain the higher cancer incidence in CVD populations [1, 4].

There is, however, growing evidence for an even stronger link between both diseases. Recent studies have shown that CVD directly enhances cancer growth. For example, myocardial infarction or the presence of heart failure (HF) both accelerate tumor growth in mice [5, 6]. Similarly, early cardiac remodeling after thoracic aortic constriction (TAC) enhances lung, colon and breast cancer growth in mice [7, 8]. Cardiac remodeling, even in the absence of contractile dysfunction, or hypertrophy induced by overexpression of activating transcription factor 3 (ATF3) in cardiomyocytes, also enhance breast cancer growth [9, 10]. Therefore, evidence is accumulating that CVD promotes cancer progression. The underlying mechanisms are still under investigation, with some studies suggesting changes in the immune system [6]. Other studies suggest that factors secreted by the diseased heart may participate, including Serpina3, Periostin, Fibronectin and Connective tissue growth factor (Ctgf) [5, 7, 9].

In this study, the impact of angiotensin II (ANGII)-induced CVD on cancer growth in vivo was investigated, differentiating between a direct effect of ANGII on tumor cells or indirect effects secondary to CVD. Herein, we hypothesized that ANGII-induced CVD would suffice to induce cancer growth.

ANGII, the main effector of the renin-angiotensin-aldosterone-system (RAAS), plays a physiological role in the homeostasis of blood pressure, and in autoregulation of glomerular filtration. ANGII is also upregulated in many CVD, for instance post-myocardial infarction, during TAC or in HF, contributing to disease progression through adverse left ventricle (LV) remodeling involving interstitial fibrosis and cardiomyocyte hypertrophy [11, 12]. Furthermore, ANGII has been implicated in cancer development promoting cancer progression by enhancing proliferation, inflammation, and angiogenesis through activation of the angiotensin II type I receptor (AT1) receptor. It is also involved in tumor microenvironment remodeling by enhancing fibroblast activation, immune cell recruitment, and pro-tumorigenic cytokine production [13, 14].

To test our hypothesis, we induced LV hypertrophy and cardiac fibrosis via ANGII infusion and investigated whether this resulted in accelerated cancer growth. Interestingly, we observed that ANGII-induced CVD alone is not sufficient to increase tumor growth, and that direct effects of ANGII on tumor cells are required to increase tumor growth.

## Methods

### Animals and ethical approval

We used ten-week-old male C57BL/6J mice (Charles River, France), as well as male and female C57BL/6J-ApcMin/J (APC_min_*)* mice (Jackson laboratory, Bar Harbor, ME, USA). All mice were housed in the animal facility of the University of Antwerp in standard cages with 12- to 12-h light/dark cycles and *ad libitum* access to regular chow and water. The Ethical Committee of the University of Antwerp approved all experiments, which conformed to Directive 2010/63/EU, the ARRIVE guidelines, and the Guide for the Care and Use of Laboratory Animals published by the US National Institutes of Health (NIH Publication no. 85– 23, revised 1996). In all experiments, mice were euthanized by intraperitoneal injection of a single dose of sodium pentobarbital (200 mg/kg; Sanofi, Belgium).

### Cell culture

Prior to injection, Lewis lung carcinoma (LLC) cells (ATCC, CRL-1642) were cultured in DMEM (Gibco, 41965-039) containing 10%-fetal calf serum (FBS, innoprot) and 1%-penicillin streptomycin (P/S, Fisher scientific, 15140122). MC38 cells (kindly provided by Prof. Sophie Lucas, Université Catholique de Louvain) were cultured in RPMI 1640 (Gibco, 11875093) containing 10%-FBS, 1%-P/S. Cells were kept at 37 °C and 5% CO_2_.

To evaluate the effect of ANGII and losartan on LLC proliferation, LLC cells (1*10^5^) cells were seeded on a 12-well plate and incubated overnight. Cells were then serum starved (0.1%-FBS) for 24 h, after which they were stimulated with either ANGII (10 µg/mL), losartan (0.1 mg/mL) or both. After 24 h, cell number was counted using the trypan blue assay (Thermo- Fisher, C10228) and an automated cell counter (Countess^®^ II).

### qPCR

To quantify differences in AT1 and angiotensin II type 2 receptor (AT2) expression between LLC and MC38 tumor cells qPCR was performed. LLC and MC38 cells were lysed and RNA extracted using the Nucleospin RNA kit (Macherey-Nagel, 740955.250) and corresponding protocol. qPCR was performed using TaqMan Fast Advanced Master Mix Kit (Applied Biosystems™ ) to determine mRNA expression of AT1, AT2 and β-actin using TaqMan probes (Thermo Fisher Scientific, cat#4304437).

### In vivo study design

#### LLC or MC38 tumor growth in ANGII-induced CVD

Osmotic mini pumps (RWD, 1004W) were subcutaneously implanted in the left flank of male C57BL/6J mice releasing ANGII at a rate of 2000 ng.kg^− 1^.min^− 1^ for four weeks. Sham operated mice were used as control receiving the same surgical procedure without the insertion of the osmotic minipump. LLC cells (5*10^5^) were injected in the right flank three weeks after starting ANGII treatment. Similar experiments were performed with injection of MC38 colon cancer cells (2*10^5^). Studies in which LLC tumor cells were injected later, i.e. 24 h after halting ANGII administration, were also performed.

Using a digital caliper, tumor size was measured every 2 days starting at day 10 until euthanasia at day 22. Tumor volume was calculated using the formula: Tumor volume = (Length*(width^2^))/2 [7]. Cardiac ultrasound imaging of the left ventricle was performed as described below at week 3 of ANGII treatment and prior to euthanasia. Heart weight, normalized to body weight, and cardiac fibrosis were determined post-mortem as indicated below.

#### APC_min_ mice

APC_min_ mice, susceptible to the development of small intestinal polyps, were placed on a high 42%-fat diet (ENVIGO, MD.88137) at the age of four weeks [15]. At six weeks, osmotic mini pumps were implanted subcutaneously in the left flank of the mice releasing ANGII at a rate of 2000 ng.kg^− 1^.min^− 1^ (*N* = 10) for four weeks. Sham-operated mice were used as control (*N* = 7). A similar experiment was performed using a low-dose of ANGII (400 ng.kg^− 1^.min^− 1^) (*N* = 7 ANGII and *N* = 7 sham). At 14 weeks, mice were euthanized, the intestine dissected, cleaned, longitudinally cut and pinned to a wax underground. Pictures were taken using the Dinocapture 2.0 camera. Polyp number was quantified manually using the FIJI software program. Cardiac function was assessed by cardiac ultrasound imaging of the left ventricle three weeks after the start of ANGII treatment and prior to euthanasia. Normalized heart weight and cardiac fibrosis were determined post-mortem as indicated below.

### Echocardiography

Ultrasound imaging was performed in anaesthetized mice on the Vevo F2 LAZR-X imaging station (Visual sonics – FUJIFILM), which was heated to 37 *±* 1 °C to stabilize body temperature of the animals and enabled recording of physiological parameters including heart rate and breathing rate, which were continuously monitored. Anesthesia was induced at 3% (v/v) and maintained at 1.5–2.5% (v/v) isoflurane (Forene; AbbVie, Wavre, Belgium). Echocardiographic images were acquired with a 57 MHz transducer (Visual Sonic) in parasternal long and short axis views. Using M-mode, images were obtained for measurement of the left ventricle posterior wall (LVPW) thickness, interventricular septum thickness (IVS) and left ventricle internal diameter (LVID) in both diastole and systole. Additionally, Fractional shortening (FS), LV-vol in diastole and systole and LV-mass were measured.

All were calculated using Vevo LAB Software (Version 3.2.0, Visual Sonics, Toronto, Canada). Lastly, LV-Mass index (LVMI) was calculated using the following formula: LVMI = LV-Mass / Body weight [16]. Echocardiography measurements were performed at 3 weeks of ANGII administration and prior to euthanasia in all experiments.

### Histology

Heart tissues were fixed in 4% formaldehyde, buffered pH = 7 (Merck, Overijse, Belgium) for 24 h, dehydrated overnight in 60% isopropanol, and then embedded in paraffin. Cardiac fibrosis was visualized using Masson’s trichrome staining a previously reported [17]. Percentage of fibrosis was calculated as total fibrotic area versus total cardiac tissue area using the FIJI analysis software. All Images were acquired with Universal Graph 6.1 software using an Olympus BX40 microscope and quantified using ImageJ.

### Cancer databases

AT1 copy number and candesartan sensitivity for human cancer cell lines were obtained from the Depmap Portal database (https://depmap.org/portal) [18]. Copy number variation (CNV) was determined by calculating the percentage of cancer cell lines that gained or lost at least one AT1 gene copy number. AT1 CNV from human cancer patients was obtained from the National cancer institute GDC data portal (https://portal.gdc.cancer.gov/), generated as part of the Cancer Genome Atlas (TCGA) Research Network (http://cancergenome.nih.gov/) and visualized based on primary cancer site.

### Data analysis and statistics

For analysis of all data Prism 9.0 (GraphPad Software, La Jolla, CA, USA) was used. Results are expressed as mean ± SEM, with N representing the number of mice. For tumor volume over time, between group differences were assessed by a two-way ANOVA test. In between group differences for echocardiography, cardiac fibrosis, heart weight, post-mortem tumor volume, weight and APC_min_ polyp count were evaluated by two-tailed T-test. Statistical significance was defined as *p* ≤ 0.05. Differences between LLC cell numbers when treated with ANGII or losartan were assessed using a Kruskal Wallis test. FC ΔCt was determined to assess differences in AT1, AT2 gene expression between LLC and MC38 tumor cells. A Mann-Whitney test was performed to assess significance in expression levels.

## Results

**Fig. 1:**
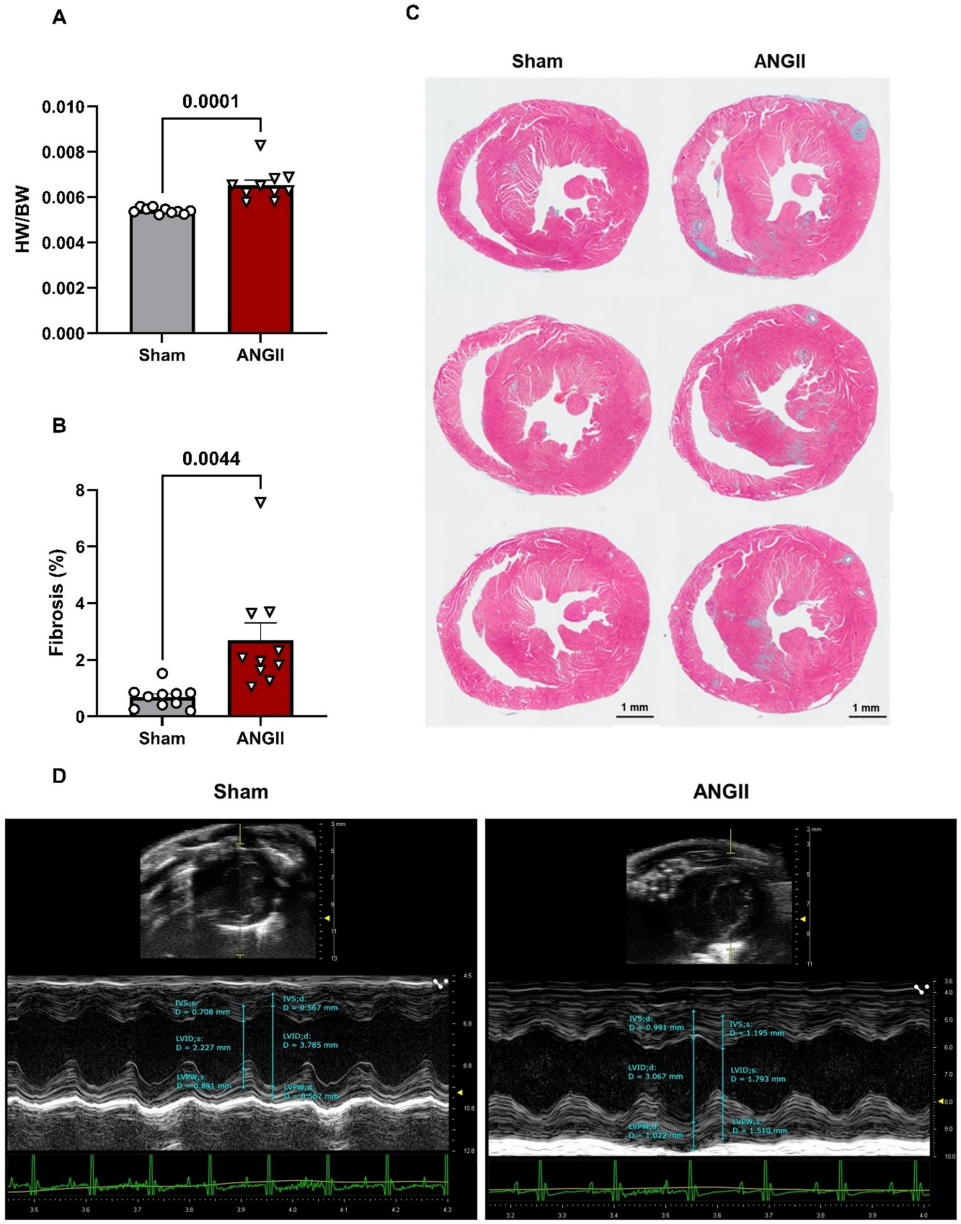
High dose ANGII (2000 ng.kg^-1^.min^-1^) induces cardiac hypertrophy and fibrosis (Sham *N* = 10, ANGII *N* = 10). **A** Normalized heart weight. **B** Masson’s trichome quantification of total cardiac fibrosis. **C** Representative images of the Masson’s trichome fibrosis staining for total cardiac fibrosis. **D** Representative echocardiography images on day 20 of ANGII treatment. Data shows mean *±* SEM. **A-B** Two-tailed T-test. HW/BW; heart weight/body weight

The aim of this study was to investigate cancer growth induced by CVD, caused by ANGII infusion. First, the ANGII infusion model was validated for the presence of CVD, in terms of LV remodeling by echocardiography and cardiac fibrosis. Mice received 28 days of ANGII administration at a concentration of 2000 ng.kg^− 1^.min^− 1^ and were euthanized at day 42, two weeks after ANGII treatment was halted. Echocardiography was performed on day 20 and 41 (prior to euthanasia) to assess LV systolic function and hypertrophy. At day 20 IVS and LVPW thickness were significantly increased both in diastole and systole in ANGII treated mice, with LVID subsequently decreasing. Systolic function was preserved as observed by the FS. Additionally, LVMI increased. Similar observations were made at day 42 (Table 1). Hence, ANGII-treated mice developed significant degrees of LV hypertrophy without changes in FS after 20 days of ANGII treatment, which persisted throughout the duration of the experiment. The presence of LV hypertrophy was confirmed by the increased normalized heart weights post-mortem (Fig. 1A). Masson’s trichrome staining also showed significantly increased cardiac fibrosis (Fig. 1B-C).

**Table 1:** Cardiac ultrasound parameters at day 20 and 41 after start of high dose ANGII

	DAY 20			DAY 41		
	Sham	ANGII	*P*-value	Sham	ANGII	*P*-value
IVS; d (mm)	0.6 *±* 0.02	1.1 *±* 0.03	< 0.0001	0.6 *±* 0.03	0.1 *±* 0.06	< 0.0001
IVS; s (mm)	0.7 *±* 0.03	1.3 *±* 0.04	< 0.0001	0.8 *±* 0.03	1.3 *±* 0.09	0.0001
LVPW; d (mm)	0.7 *±* 0.02	1.1 *±* 0.05	< 0.0001	0.7 *±* 0.02	1.0 *±* 0.05	0.0001
LVPW; s (mm)	0.7 *±* 0.02	1.3 *±* 0.04	< 0.0001	0.9 *±* 0.04	1.3 *±* 0.08	0.001
LVID; d (mm)	4.5 *±* 0.1	3.3 *±* 0.2	< 0.0001	4.4 *±* 0.09	4.1 *±* 0.2	0.1
LVID; s (mm)	3.6 *±* 0.2	2.3 *±* 0.2	< 0.0001	3.5 *±* 0.1	3.1 *±* 0.2	0.08
LV-Mass (mg)	84.2 *±* 3.1	103.3 *±* 6.1	0.009	85.3 *±* 5.3	133.1 *±* 14.0	0.005
LVMI (mg/g)	3.0 *±* 0.1	3.7 *±* 0.2	0.01	3.0 *±* 0.2	4.7 *±* 0.5	0.004
FS (%)	19.6 *±* 2.2	31.5 *±* 3.3	0.007	20.4 *±* 1.6	24.6 *±* 2.5	0.2
LV-Vol; d (µl)	94.1 *±* 5.1	45.7 *±* 5.7	< 0.0001	86.4 *±* 4.1	73.8 *±* 6.8	0.1
LV-vol; s (µl)	57.3 *±* 5.1	19.6 *±* 3.8	0.02	51.2 *±* 4.5	38.9 *±* 4.7	0.08
Heart rate (BPM)	444 *±* 16	436 *±* 12	0.7	435 *±* 16	490 *±* 14	0.02

Data show mean *±* SEM. P-values were determined using Two-tailed T-test

*IVS* Intra ventricle septum thickness in diastole (d) and systole (s), *LVID* Left ventricle internal diameter, *LVPW* Left ventricle posterior wall thickness, *LVMI* left ventricle mass index, *FS* Fractional shortening and *LV-vol* left ventricle volume in d and s

**Fig. 2:**
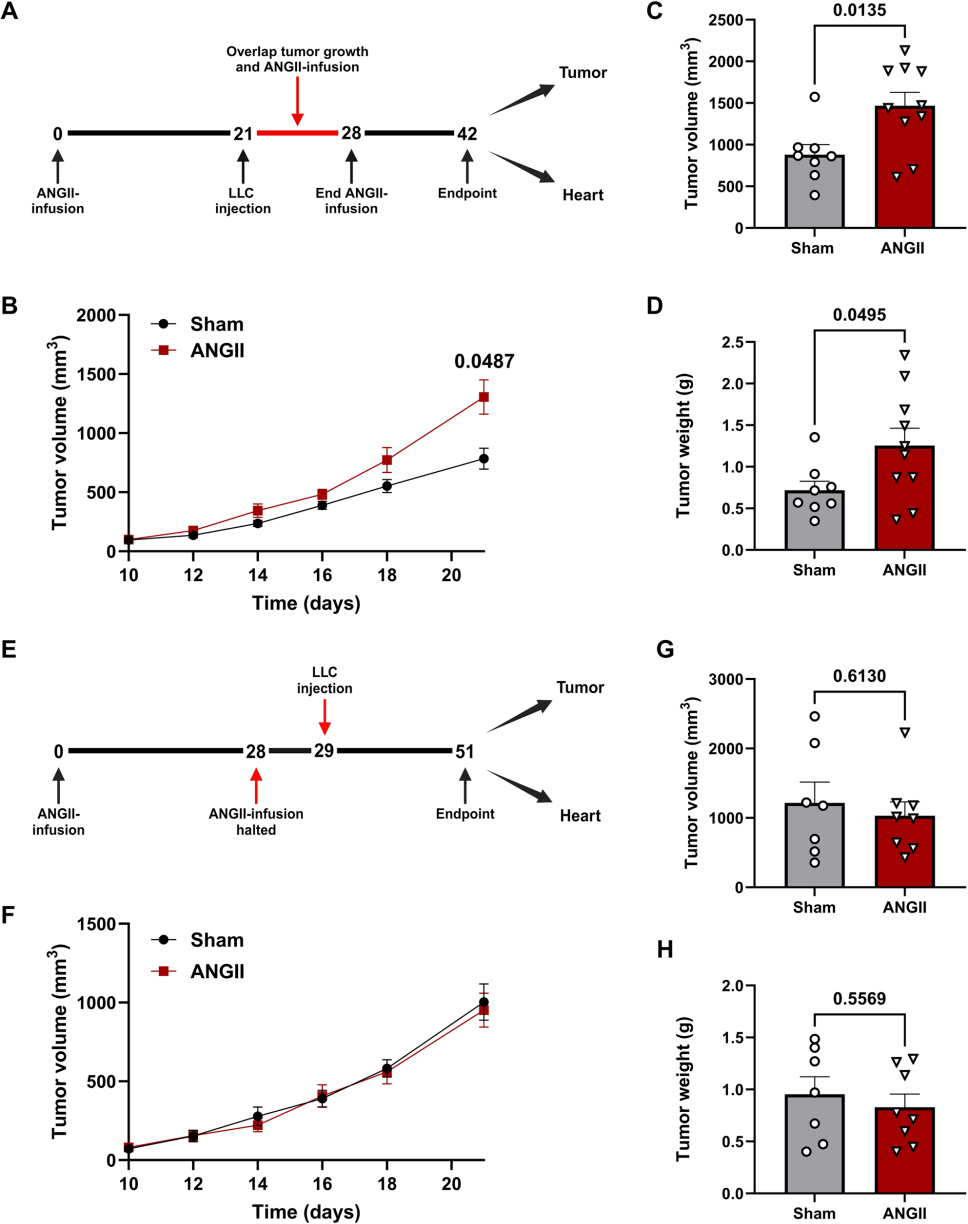
LLC tumor growth with and without overlap of ANGII administration. **A** Experimental design of the ‘ANGII overlap experiment’ (Sham *N* = 8, ANGII *N* = 10). **B** LLC tumor volume over time, **C-D** tumor volume and weight post-mortem, respectively. **E** Experimental design of the ‘without ANGII overlap experiment’ (Sham *N* = 7, ANGII *N* = 8). **F** LLC tumor volume over time. **G-H** Tumor volume and weight post-mortem. Data shows mean *±* SEM. **B**,** H** Two-way ANOVA test. **C-D**,** G-H** Two-tailed T-test

Secondly, cancer growth was evaluated in this model. Mice were injected with LLC cells 21 days after the start of ANGII administration, hence including a one week overlap between ANGII administration and LLC cancer growth (Fig. 2A). Using this protocol, tumor growth was significantly accelerated in ANGII treated mice compared to the sham control group. Importantly, the growth curves between sham control and ANGII-treated mice started to diverge early, upon the first measurements of tumor volume (Fig. 2B). Post-mortem tumor volume and weight after 21 days of growth confirmed increased tumor growth in ANGII treated mice (Fig. 2C and D).

**Fig. 3:**
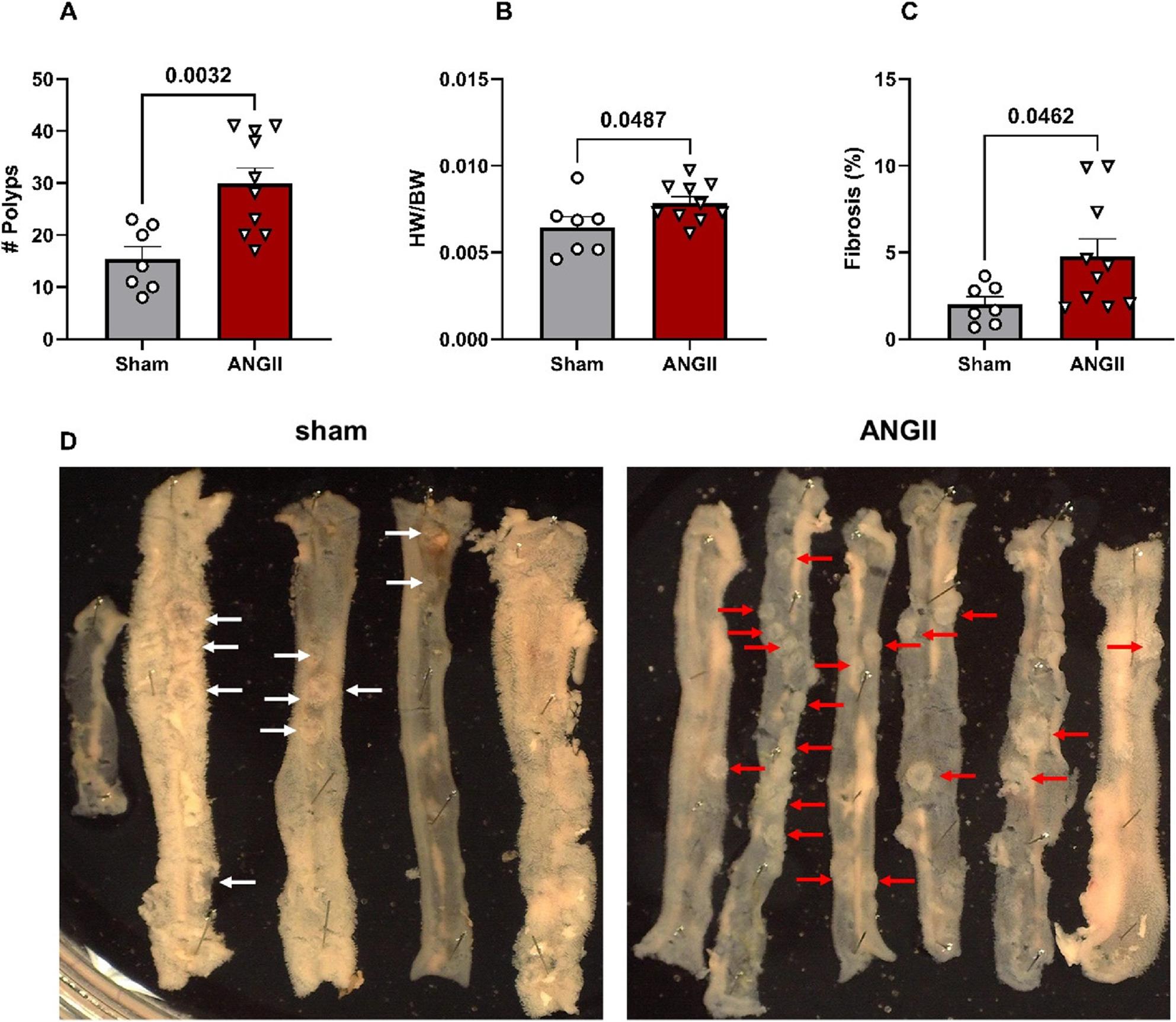
Polyp development in APC_min_ mice treated with a high dose of ANGII (Sham *N* = 7, ANGII high dose *N* = 10). **A** Polyp count, **B** normalized heart weight, **C** cardiac fibrosis, **D** representative image of polyp growth in the small intestine for the distinct experimental conditions. Arrows indicate more distinctly formed polyps. Data represent mean + SEM. **A-C** Two-tailed T-test. HW/BW; heart weight to body weight

Next, we tested whether this effect was reproducible when ANGII administration was halted prior to LLC cancer cell injection, hence avoiding overlap between ANGII administration and cancer growth (Fig. 2E). ANGII infusion was halted after 28 days and LLC cells injected 24 h later. Using this protocol, no significant difference in tumor growth was observed (Fig. 2F-H). In both experiments, echocardiography—performed at week three of ANGII treatment and prior to euthanasia—demonstrated the presence of cardiac hypertrophy throughout the duration of the experiments, which was further supported by significantly increased cardiac fibrosis and normalized heart weights (supplemental Tables 1 and 2, supplemental Figs. 1 and 2). These data, therefore, indicate that an overlap between ANGII infusion and the presence of cancer cells was necessary to trigger increased tumor growth, suggesting an effect of ANGII on the tumor cells, rather than an indirect effect secondary to CVD. To challenge the hypothesis that ANGII directly impacts cancer growth, rather than the presence of ANGII-induced CVD, we used another cancer model, specifically the APC_min_ model, which has previously been shown to develop a higher cancer burden after TAC induced cardiac remodeling and in the presence of HF [5, 8]. First, polyp growth was assessed after high dose administration of ANGII (2000 ng.kg^− 1^.min^− 1^). APC_min_ mice were treated with ANGII from six to ten weeks old. Mice were euthanized at 14 weeks, and the intestinal polyps were quantified. ANGII treated mice had a significantly higher polyp count compared to the sham control group (Fig. 3A).

**Fig. 4:**
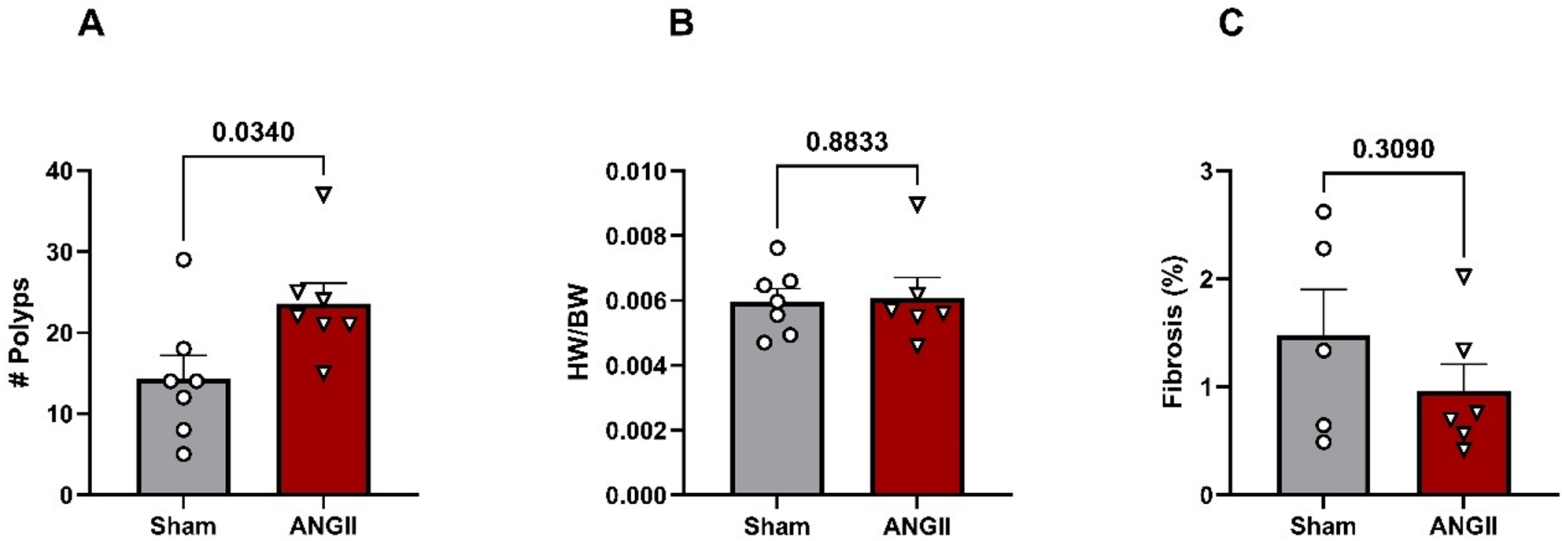
Polyp development in APC_min_ mice treated with a low dose of ANGII (Sham *N* = 7, ANGII low dose *N* = 7). **A** Polyp count, **B** normalized heart weight, **C** cardiac fibrosis. Data represent mean *±* SEM. **A-C** Two-tailed T-test. HW/BW; heart weight to body weight

Next, to differentiate between directs effects of ANGII or indirect effects secondary to CVD a similar experiment using a low-dose of ANGII (400 ng.kg^− 1^.min^− 1^) was performed. Low-dose ANGII treatment resulted in increased polyp count (Fig. 4A) similar to high dose ANGII treatment, however, whereas high dose ANGII treatment induced LV hypertrophy and cardiac fibrosis (supplemental Table 3) low dose ANGII infusion, consistent with previous studies, did not [19, 20]. Cardiac ultrasound measurements showed no differences in IVS, LVPW, LVID or LVMI parameters between ANGII-treated and control animals (Table 2). Additionally, normalized heart weight and fibrosis levels did not differ between ANGII–treated and control mice (Fig. 4B, C). Together these results, therefore, support the hypothesis that ANGII stimulates (APC_min_) cancer development directly rather than indirectly.

**Table 2:** Cardiac ultrasound parameters for APC_min_ mice at day 20 and 56 after the start of low-dose ANGII (400 ng.kg^− 1^.min^− 1^) treatment

		DAY 20			DAY 56			
		Sham	ANGII	*P*-value	Sham		ANGII	*P*-value
IVS; d (mm)		0.5 *±* 0.03	0.5 *±* 0.01	0.6	0.5 *±* 0.03		0.6 *±* 0.03	0.1
IVS; s (mm)		0.5 *±* 0.04	0.5 *±* 0.02	0.5	0.7 *±* 0.09		0.7 *±* 0.05	0.8
LVPW; d (mm)		0.5 *±* 0.04	0.5 *±* 0.01	0.9	0.7 *±* 0.07		0.7 *±* 0.08	0.9
LVPW; s (mm)		0.6 *±* 0.03	0.6 *±* 0.02	0.2	0.8 *±* 0.1		0.8 *±* 0.08	0.9
LVID; d (mm)		4.1 *±* 0.08	4.2 *±* 0.06	0.4	4.1 *±* 0.1		4.3 *±* 0.1	0.3
LVID; s (mm)		3.6 *±* 0.07	3.7 *±* 0.06	0.08	3.5 *±* 0.2		3.8 *±* 0.1	0.2
LV-Mass (mg)		52.8 *±* 4.8	52.8 *±* 1.9	0.99	68.2 *±* 5.5		82.0 *±* 8.3	0.2
LVMI (mg/g)		3.5 *±* 0.2	4.4 *±* 0.1	0.7	2.8 *±* 0.5		3.0 *±* 0.4	0.7
FS (%)		13.8 *±* 1.5	11.3 *±* 0.5	0.1	16.1 *±* 3.7		12.7 *±* 1.3	0.4
LV-Vol; d (µl)		75.5 *±* 3.3	79.1 *±* 2.5	0.4	75.3 *±* 4.3		85.2 *±* 6.7	0.3
LV-vol; s (µl)		53.0 *±* 2.6	59.6 *±* 2.2	0.08	51.0 *±* 6.9		62.0 *±* 5.4	0.2
Heart rate (BPM)		464 *±* 22	497 *±* 15	0.2	500 *±* 16		521 *±* 15	0.4

Data show mean *±* SEM. P-values were determined using Two-tailed T-test

*IVS* Intra ventricle septum thickness in diastole (d) and systole (s), *LVID* left ventricle internal diameter, *LVPW* left ventricle posterior wall thickness, *LVMI* left ventricle mass index, *FS* fractional shortening and *LV-vol* left ventricle volume in d and s

**Fig. 5:**
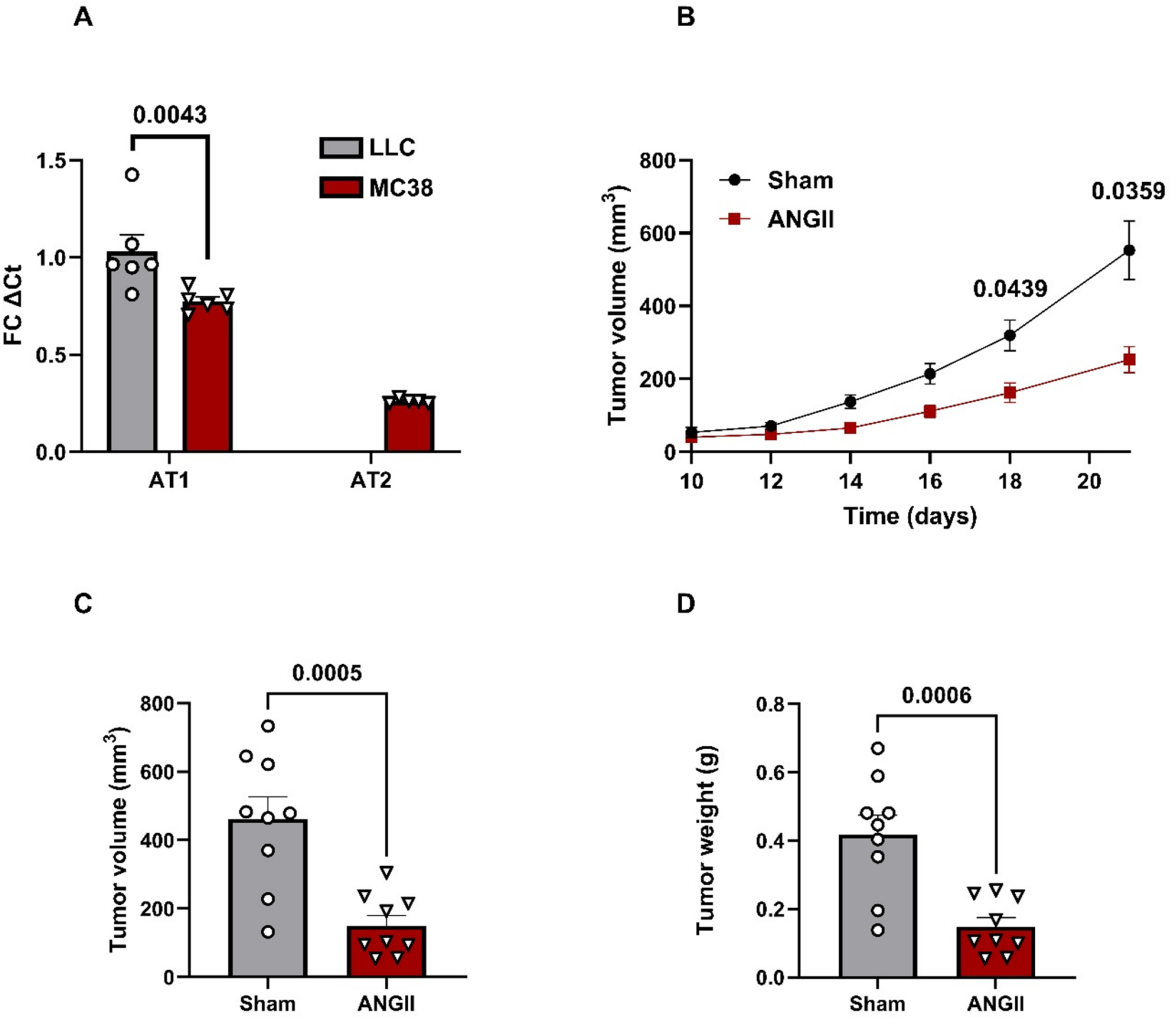
MC38 tumor growth when concomitantly treated with ANGII (Sham *N* = 9, ANGII *N* = 9). **A** Differences in AT1 and AT2 mRNA expression in LLC and MC38 tumor cells quantified by qPCR. All expression values are shown relative to LLC AT1 expression. AT1 expression in MC38 tumor cells was significantly lower compared to LLC tumor cells. **B** Tumor growth over time. **C-D** tumor volume and weight post-mortem, respectively. Data represents mean *±* SEM. **A** Mann-Whitney test, **B** Two-way ANOVA, **C-D** Two-tailed T-test

Lastly, an experiment was performed using MC38 colon cancer cells and the protocol including overlap between high dose ANGII administration and tumor growth. MC38 tumor cells have low expression of the AT1 receptor in comparison to LLC tumor cells as illustrated by Fig. 5A. Interestingly, MC38 tumor growth was significantly slower in the ANGII treated group (Fig. 5B-D), in contrast to LLC tumor growth under similar conditions.

**Fig. 6:**
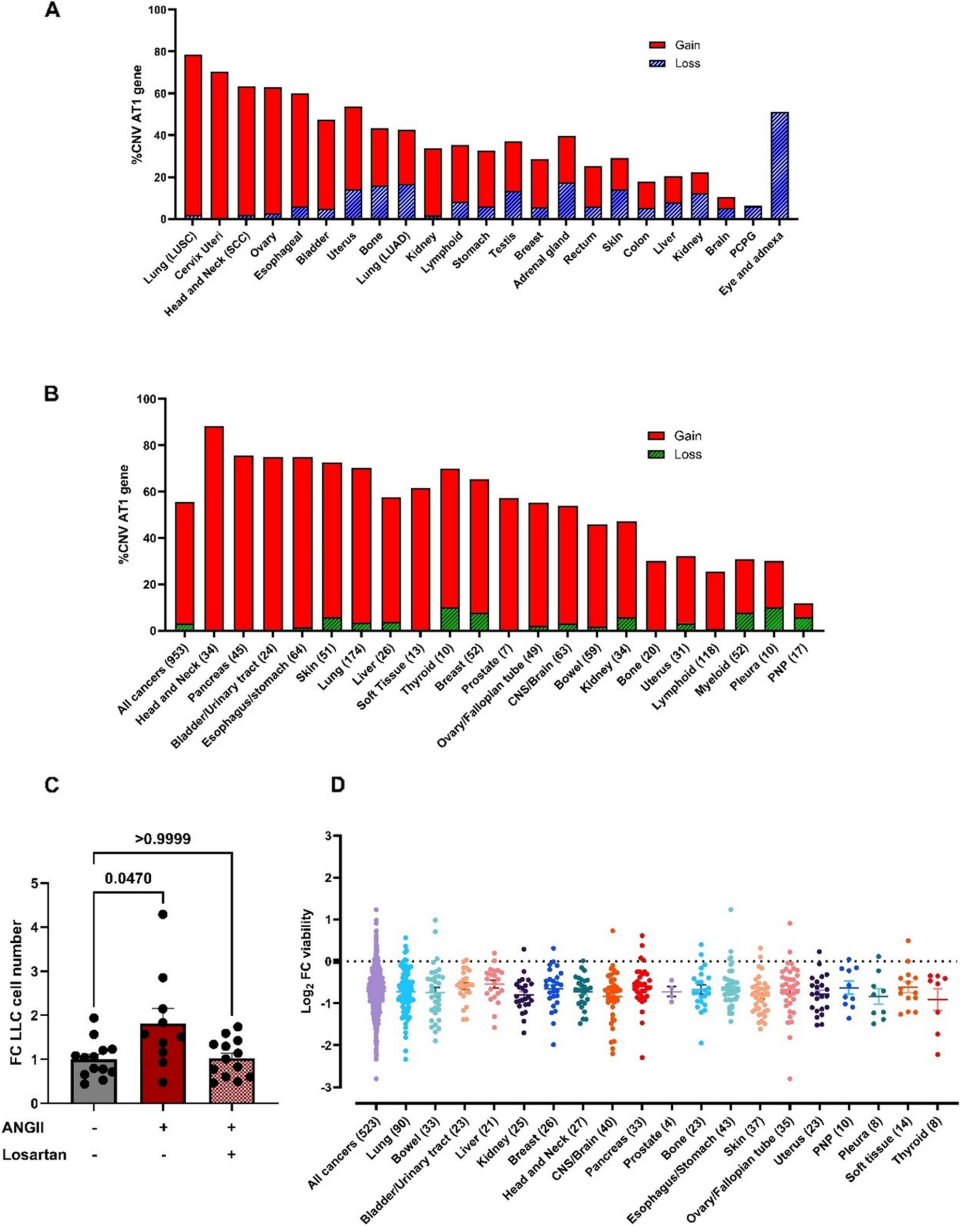
Percentage of CNV in patient tumors and human cancer cell lines. **A** Percentage of AT1 CNV in patient tumors as determined by the TCGA database. **B** Percentage of AT1 CNV in human cancer cell lines. Copy number gain or loss was set as a minimal of one copy number gain or loss. Numbers in parentheses indicate the number of cell lines per cancer type. **C** LLC cell number when treated with ANGII (10 µg/mL) and/or losartan (0.1 mg/mL) in vitro (*N* = 2). **D** Candesartan sensitivity of human cancer cell lines in the Depmap database. Numbers in parentheses indicate the number of cell lines per cancer type. **C-D** Data show Mean *±* SEM. **C** Kruskal Wallis test. LUSC; Lung squamous cell carcinoma, SCC; squamous cell carcinoma, LUAD; Lung adenocarcinoma, PCPG; Pheochromocytoma and Paraganglioma, CNS; Central nervous system, PNP; Peripheral nervous system

The data above show that ANGII may directly enhance tumor growth, depending on AT1 receptor the expression. We therefore evaluated AT1 CNV in patient tumors and human cancer cell lines using data obtained from the TCGA and Depmap portal databases, respectively. Cancer cells can regulate gene expression by changing the number of copies from a particular gene benefiting cancer development. We found that human tumors displayed both gain and loss of AT1 copy number, but with a higher propensity to a gain of AT1 copy number (Fig. 6A). When assessing CNV in human cancer cell lines similar results were found (Fig. 6B). The role of the AT1 receptor was further evaluated in vitro by treating LLC cells with ANGII (10 µg/mL) and/or AT1 blocker losartan (0.1 mg/mL). ANGII treatment increased LLC cell proliferation significantly compared to the control group. Conversely, this effect was not observed in the presence of both losartan and ANGII (Fig. 6C). In addition, the impact of AT1 blocker, specifically candesartan, on human cancer cell line viability was explored using data from the Depmap portal database. These data show that cell viability decreases upon exposure to candesartan for the large majority of cancer cell lines (Fig. 6D). Collectively these data further support our hypothesis that ANGII can directly impact tumor growth via the AT1 receptor.

## Discussion

Historically, an increased incidence of cancer in CVD populations has been explained by shared risk factors and common pathophysiological pathways. Recent studies suggest a stronger link between both diseases, with CVD directly enhancing cancer growth. Several models of CVD have been explored in mice, including TAC induced cardiac hypertrophy, arterial hypertension and HF [5, 9, 10]. These studies have led to the hypothesis that CVD causes the secretion of growth factors by the diseased heart or results in immune modulation, which may accelerate cancer growth. In the present study, using different types of cancer and cancer models, we show that ANGII, the main effector of RAAS and an important factor in the progression of CVD, may directly contribute to enhanced tumor growth when the cancer cells abundantly express the AT1 receptor.

The conclusion of this study that ANGII directly impacted tumor growth was supported by three fundamental experimental observations. First, using the ANGII infusion model, we observed that enhanced growth of LLC cancer cells only occurred when applying an overlap between ANGII infusion and the presence of LCC cells. When injecting the LCC cells after halting ANGII infusion, growth of LCC cells was not affected by ANGII treatment. Second, using the APC_min_ genetic colon cancer mice model even a low dose of ANGII, which did not increase cardiac fibrosis or induce LV-hypertrophy, resulted in significantly more intestinal polyps. Third, ANGII treatment did not enhance the growth of MC38 tumors, a cancer cell line that has low AT1 expression as opposed to the LLC cancer cells. Instead, MC38 tumors showed reduced tumor growth following ANGII exposure, indicating a differential response when compared with LLC and APC_min_ tumors. Interestingly, AT2 expression was observed in MC38 but not in LLC tumor cells. Previous studies have shown AT2 activation to be able to attenuate cancer growth [14, 21, 22]. The reduction in MC38 tumor growth might therefore be attributed to the presence of the AT2 receptor.

ANGII activation of AT1 has previously been linked to pro-tumorigenic processes, including cell proliferation, angiogenesis, inflammation, and apoptotic resistance [14]. Moreover, the release of ANGII stimulates production of vascular endothelial growth factor and other pro-angiogenic cytokines, promoting neovascularization essential for tumor growth [23, 24]. It also activates intracellular signaling pathways such as MAPK/ERK, PI3K/Akt, and NF-κB, which support cell survival, migration, and invasion [25–27]. Additionally, ANGII increases oxidative stress subsequently contributing to an inflammatory microenvironment through the upregulation of reactive oxygen species and inflammatory mediators like IL-6 and TNF-α, further enhancing tumor progression [28, 29]. These studies support ANGII as a tumor-promoting factor.

Notably, the high-dose of ANGII (2000 ng·kg⁻¹·min⁻¹) used in this study may result in ANGII plasma levels that could exceed endogenous pathological levels in mice. In contrast, the low concentration of ANGII (400 ng·kg⁻¹·min⁻¹) used in the APC_min_ mice might better reflect endogenous pathophysiological ANGII concentrations in mice. Furthermore, reports have also shown the ANGII model to be an inducer of Periostin, a factor that has been observed to potentiate tumor growth after TAC surgery [7, 30]. Although Periostin levels were not measured, our data suggest that Periostin induction alone is insufficient to enhance tumor growth in this model, and that ongoing ANGII signaling drives the tumor-promoting effect.

The finding that ANGII directly stimulates cancer growth when the tumor expresses higher levels of the AT1 receptor may be highly clinically relevant, particularly for CVD patients, since ANGII also plays an important role in numerous human diseases such as HF, hypertension and chronic kidney disease [28]. This is underscored by the AT1 gene CNV in human cancer cell lines and patient tumors found in public databases. AT1 copy number is increased in the majority of cancers, despite variability among different types of cancers. Furthermore, when evaluating the effect of AT1 blocker candesartan on human cancer cell line viability, decreased viability was observed for the majority of cell lines. Moreover, the role of the AT1 receptor in our models of ANGII-induced tumor growth was further corroborated by treatment of LLC cells with ANGII and AT1 blocker losartan in vitro. Treatment of LLC cells with ANGII increased proliferation. This effect was not observed in the presence of both ANGII and losartan. Collectively, therefore, these data support the hypothesis that ANGII directly impacts tumor growth via the AT1 receptor.

The use of AT1 blockers and their impact on cancer growth has been an ongoing debate for decades. An initial report concerning lung cancer and the use of AT1 blockers indicated an increased risk of new cancer occurrence [31]. A later study conducted by the US food and drug administration (FDA) found no additional risk for the development of lung cancer, whereas other studies report a potential beneficial effect [32–35]. Similar disparities in study results can also be observed for other cancer types.

The conflicting outcomes between studies investigating the impact of AT1 blockers on cancer incidence, progression and mortality in cancer-free CVD-populations might be attributed to differences in study design, size and heterogeneity in patient population, method of analysis and inhomogeneity in the use of AT1 blockers. In addition, clinical studies investigating the impact of AT1 blockers in cancer populations are scarce, although some pre-clinical studies have reported cancer attenuating effects [14]. Moreover, some studies have shown a link between expression levels of AT1 in tumors and patient outcome, with higher AT1 expression being associated with a worse prognosis [24, 36, 37]. Additionally, a study in breast cancer patients reported a subpopulation of ER-positive, ERBB2 negative breast cancer tumors with overexpression of AT1. Furthermore, overexpression of AT1 in H16N2 mammary epithelial cells, when combined with ANGII dosing, resulted in a highly invasive phenotype, which was attenuated by the AT1 receptor blocker losartan. Consistent with this, losartan was shown to reduce tumor growth in MCF7 breast cancer xenografts overexpressing AT1 [38]. Together, these studies suggest AT1-inhibition in those tumors highly expressing AT1 might have beneficial effects.

The results of our study corroborate a potential therapeutic role for AT1 blockers in cancer, particularly in tumors abundantly expressing the AT1 receptor and in the presence of co-morbidities where RAAS is activated, hence warranting further clinical research and the need for controlled clinical studies investigating the use of AT1 blockers in cancer.

Furthermore, the observation that MC38 tumors grow more slowly in mice treated with ANGII is interesting and not reported previously. These data suggest that RAAS activation can have both a positive and negative impact on tumor growth. Further research is required to study the effect of ANGII on distinct tumor types and the underlying causes of tumor reduction. This would contribute to a better understanding of the link between CVD and cancer. Subsequently, this might lead to new insights potentially improving treatment options of both cancer and CVD, particularly in patients that have both diseases concurrently.

In conclusion, our data indicate that direct effects of ANGII on cancer cells may promote cancer growth, warranting further investigation into an activated RAAS as a potential mechanism linking CVD and enhanced cancer growth. Although more research is required, our findings suggest that cancer patients, particularly those with comorbid conditions inducing RAAS activation, might benefit from diagnostic analysis of AT1 receptor abundancy in tumors, and treatment with AT1 receptor blockers.

### Limitations

We have previously characterized the ANGII infusion model including the high and low dose of ANGII infusion, but have not measured blood pressure in this study [20, 39, 40]. Furthermore, the high ANGII dose used in this study (2000 ng·kg⁻¹·min⁻¹) may generate circulating ANGII concentrations that exceed endogenous pathological levels in mice and therefore may reflect more extreme conditions. Moreover, further studies employing AT1 receptor blockers, or manipulating AT1 expression in LLC or MC38 cells, would strengthen the conclusions of this work. Lastly, although no cardiac remodeling or injury was detected in APC_min_ mice treated with low‑dose ANGII, early‑stage remodeling markers such as Serpina3 or Periostin were not assessed. The possibility of subtle cardiac changes contributing to intestinal polyp growth can therefore not be fully excluded.

## Supplementary information

Supplementary Material 1.

## Data Availability

All data generated or analyzed during this study are included in this published article (and its supplementary information files).
