# Supplementary material for: The role of angiotensin II in cardiovascular disease-induced cancer growth
Source: Cardiooncology. 2026 May 1;12:79. doi: 10.1186/s40959-026-00495-x (PMC13277031; doi:10.1186/s40959-026-00495-x)
Supplement: Supplementary file 1 — Supplementary Material 1. [file 40959_2026_495_MOESM1_ESM.docx]

**Supplemental material**

**Supplemental table 1. Echocardiographic parameters of the LLC-overlap experiment (figure 2A).**

|  | DAY 20 | | | | | DAY 41 | | |
| --- | --- | --- | --- | --- | --- | --- | --- | --- |
|  | | **Sham** | **ANGII** | **P-value** | **Sham** | | **ANGII** | **P-value** |
|  | |  |  |  |  | |  |  |
| **IVS;d (mm)** | | 0.6 + 0.04 | 0.9 + 0.05 | 0.0003 | 0.5 + 0.03 | | 0.8 + 0.02 | <0.0001 |
| **IVS’s (mm)** | | 0.8 + 0.07 | 1.2 + 0.07 | 0.001 | 0.7 + 0.05 | | 1.1 + 0.05 | <0.0001 |
| **LVPW;d (mm)** | | 0.7 + 0.04 | 0.9 + 0.03 | 0.002 | 0.7 + 0.03 | | 0.9 + 0.04 | 0.004 |
| **LVPW;s (mm)** | | 1.0 + 0.1 | 1.3 + 0.05 | 0.03 | 0.9 + 0.05 | | 1.2 + 0.07 | 0.002 |
| **LVID;d (mm)** | | 4.2 + 0.2 | 3.9 + 0.1 | 0.1 | 4.3 + 0.2 | | 4.5 + 0.2 | 0.7 |
| **LVID;s (mm)** | | 3.2 + 0.3 | 2.7 + 0.2 | 0.2 | 3.3 + 0.3 | | 3.2 + 0.3 | 0.8 |
| **LV-Mass (mg)** | | 80.2 + 5.2 | 105.6 + 6.1 | 0.007 | 78.1 + 6.3 | | 122.0 + 9.0 | 0.002 |
| **LVMI (mg/g)** | | 3.0 + 0.2 | 3.8 + 0.2 | 0.001 | 2.90 + 0.3 | | 4.3 + 0.3 | 0.002 |
| **FS (%)** | | 26.3 + 4.5 | 30.1 + 3.1 | 0.5 | 24.0 + 2.8 | | 29.3 + 3.1 | 0.2 |
| **LV-Vol;d (µl)** | | 81.1 + 8.0 | 65.6 + 5.6 | 0.1 | 87.0 + 10.4 | | 92.6 + 9.2 | 0.7 |
| **LV-vol;s (µl)** | | 42.7 + 7.2 | 29.2 + 4.7 | 0.1 | 47.2 + 7.8 | | 45.3 + 9.0 | 0.9 |
| Data show mean + SEM. P-values were determined using Two-tailed T-test. LVID, left ventricle internal diameter, LVPW, left ventricle posterior wall thickness and IVS, Intra ventricle septum thickness in diastole (d) and systole (s). LVMI, left ventricle mass index, FS, fractional shortening, LV-vol, left ventricle volume in d and s. | | | | | | | | |


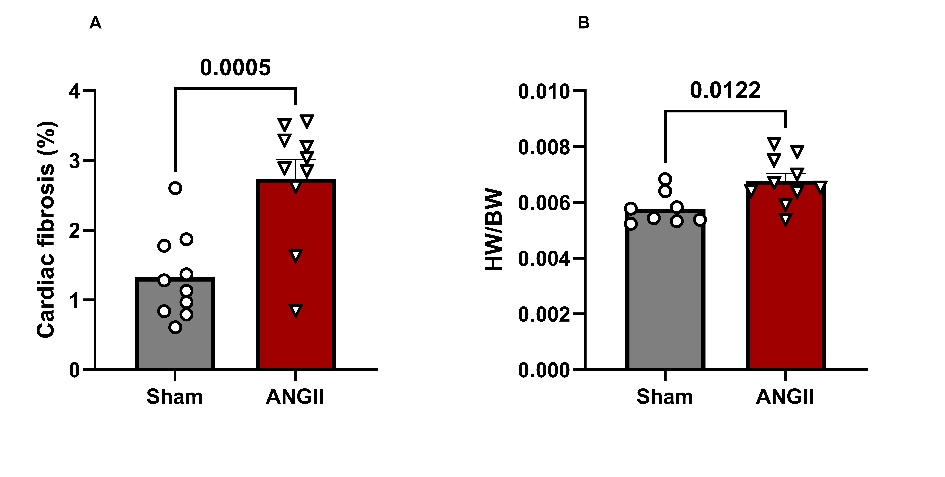


**Supplemental figure 1.** Cardiac fibrosis and normalized heart weight of the LLC-overlap experiment. **A)** Cardiac fibrosis and **B)** Normalized heart weight. **A, B)** Two-tailed T-test. HW/BW ; Heart weight to body weight.

**Supplemental table 2. Echocardiographic parameters of LLC non-overlap experiment (figure 2E).**

|  | DAY 20 | | | | | DAY 51 | | |
| --- | --- | --- | --- | --- | --- | --- | --- | --- |
|  | | **Sham** | **ANGII** | **P-value** | **Sham** | | **ANGII** | **P-value** |
|  | |  |  |  |  | |  |  |
| **IVS;d (mm)** | | 0.6 + 0.04 | 0.9 + 0.04 | 0.0001 | 0.7 + 0.03 | | 0.9 + 0.06 | 0.0003 |
| **IVS’s (mm)** | | 0.8 + 0.08 | 1.2 + 0.07 | 0.003 | 0.9 + 0.04 | | 1.2 + 0.05 | <0.0001 |
| **LVPW;d (mm)** | | 0.7 + 0.03 | 1.0 + 0.04 | 0.001 | 0.7 + 0.04 | | 0.9 + 0.02 | 0.0007 |
| **LVPW;s (mm)** | | 0.9 + 0.04 | 1.2 + 0.06 | 0.003 | 0.9 + 0.05 | | 1.1 + 0.03 | 0.01 |
| **LVID;d (mm)** | | 4.4 + 0.2 | 4.1 + 0.1 | 0.2 | 4.2 + 0.2 | | 4.5 + 0.2 | 0.3 |
| **LVID;s (mm)** | | 3.6 + 0.2 | 3.3 + 0.2 | 0.2 | 3.3 + 0.2 | | 3.6 + 0.2 | 0.3 |
| **LV-Mass (mg)** | | 91.9 + 10.1 | 123.7 + 8.0 | 0.03 | 82.6 + 9.4 | | 127.1 + 7.0 | 0.002 |
| **LVMI (mg/g)** | | 3.3 + 0.3 | 4.3 + 0.3 | 0.04 | 3.0 + 0.3 | | 4.4 + 0.3 | 0.003 |
| **FS (%)** | | 18.6 + 1.8 | 21.3 + 2.4 | 0.4 | 22.5 + 2.4 | | 19.4 + 1.6 | 0.3 |
| **LV-Vol;d (µl)** | | 87.9 + 8.3 | 75.8 + 6.3 | 0.3 | 79.4 + 8.1 | | 91.5 + 8.4 | 0.3 |
| **LV-vol;s (µl)** | | 54.7 + 6.9 | 43.7 + 5.6 | 0.2 | 45.4 + 7.6 | | 55.9 + 6.5 | 0.3 |
| Data show mean + SEM. P-values were determined using Two-tailed T-test. LVID, left ventricle internal diameter, LVPW, left ventricle posterior wall thickness and IVS, Intra ventricle septum thickness in diastole (d) and systole (s). LVMI, left ventricle mass index, FS, fractional shortening, LV-vol, left ventricle volume in d and s. | | | | | | | | |


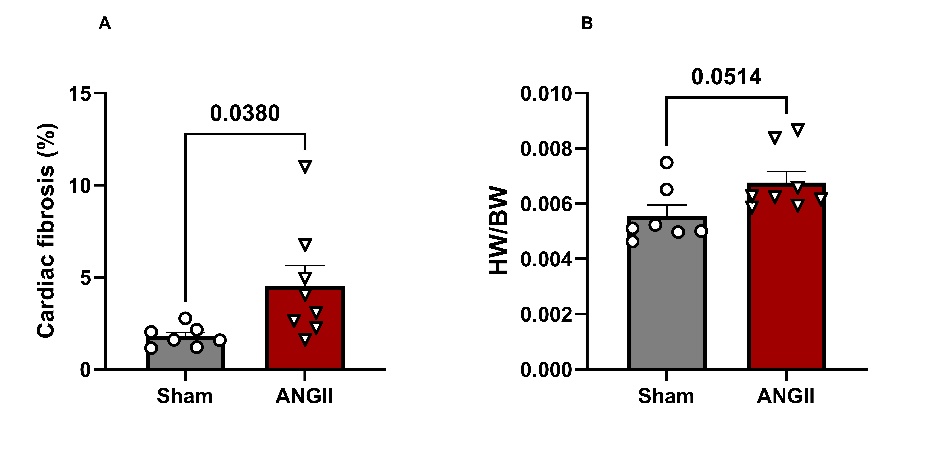


**Supplemental figure 2.** Cardiac fibrosis and normalized heart weight of the LLC non-overlap experiment. **A)** Cardiac fibrosis and **B)** Normalized heart weight. **A, B)** Two-tailed T-test. HW/BW ; Heart weight to body weight.

**Supplemental table 3. Echocardiography parameters of high dose ANGII (2000 ng.kg^-1^.min^-1^) treatment at day 20 and 56 in APC_min_ mice (figure 3).**

|  | DAY 20 | | | | | DAY 56 | | |
| --- | --- | --- | --- | --- | --- | --- | --- | --- |
|  | | **Sham** | **ANGII** | **P-value** | **Sham** | | **ANGII** | **P-value** |
|  | |  |  |  |  | |  |  |
| **IVS;d (mm)** | | 0.5 + 0.02 | 1.1 + 0.09 | 0.0008 | 0.6 + 0.08 | | 0.9 + 0.03 | 0.007 |
| **IVS’s (mm)** | | 0.6 + 0.09 | 1.5 + 0.2 | 0.003 | 0.7 + 0.09 | | 1.3 + 0.07 | 0.005 |
| **LVPW;d (mm)** | | 0.6 + 0.01 | 1.0 + 0.1 | 0.02 | 0.7 + 0.02 | | 0.9 + 0.03 | 0.007 |
| **LVPW;s (mm)** | | 0.7 + 0.05 | 1.5 + 0.2 | 0.004 | 0.9 + 0.08 | | 1.2 + 0.04 | 0.009 |
| **LVID;d (mm)** | | 3.6 + 0.2 | 2.9 + 0.2 | 0.07 | 4.0 + 0.30 | | 4.1 + 0.1 | 0.5 |
| **LVID;s (mm)** | | 2.5 + 0.3 | 1.6 + 0.3 | 0.07 | 2.9 + 0.4 | | 2.8 + 0.1 | 0.8 |
| **LV-Mass (mg)** | | 47.9 + 6.8 | 100.8 + 11.6 | 0.03 | 73.2 + 13.4 | | 119.8 + 8.2 | 0.03 |
| **LVMI (mg/g)** | | 1.8 + 0.1 | 4.4 + 0.5 | 0.007 | 3.2 + 0.5 | | 6.5 + 0.4 | 0.003 |
| **FS (%)** | | 30.1 + 4.9 | 47.7 + 6.2 | 0.07 | 27.4 + 3.7 | | 32.0 + 1.2 | 0.2 |
| **LV-Vol;d (µl)** | | 55.3 + 9.2 | 32.9 + 6.0 | 0.09 | 69.0 + 12.7 | | 76.0 + 5.2 | 0.6 |
| **LV-vol;s (µl)** | | 24.9 + 8.2 | 8.1 + 4.1 | 0.1 | 33.5 + 10.7 | | 30.1 + 2.6 | 0.7 |
| Data show mean + SEM. P-values were determined using Two-tailed T-test. LVID, left ventricle internal diameter, LVPW, left ventricle posterior wall thickness and IVS, Intra ventricle septum thickness in diastole (d) and systole (s). LVMI, left ventricle mass index, FS, fractional shortening, LV-vol, left ventricle volume in d and s. | | | | | | | | |
